# Direct physical vapor deposition and flexible photoelectrical properties of large-area free-standing films of metal octaethylporphyrin on ionic liquid surface

**DOI:** 10.1038/s41598-017-10293-2

**Published:** 2017-08-29

**Authors:** Yan Xiao, Feng-Xia Wang, Jia-Mei Yang, Miao-Rong Zhang, Ge-Bo Pan

**Affiliations:** 1 Suzhou Institute of Nano-tech and Nano-bionics, Chinese Academy of Sciences Suzhou, 215125 Jiangsu, China; 2 University of Chinese Academy of Sciences, 100049 Beijing, P.R. China

## Abstract

Free-standing films of metal octaethylporphyrins (MOEPs) were prepared for the first time by a physical vapor deposition on surface of an ionic liquid (IL). Different from those on solid surfaces, the as-obtained films were very compact and with plannar structure. The monitoring of time-dependent process indicated that the high surface energy of IL and the strong π…π interaction between MOEP molecules played key roles in forming such films. Furthermore, the as-obtained film showed good transferability, which made it possible to be easily transferred to any substrates for further device application. More importantly, the prototype photodetectors based on free-standing films of MOEP showed ultra flexibility, mechanical stability, and durability.

## Introduction

Ordered crystalline films of organic semiconductors are one of the most important elements in high-performance optical and electronic devices^1, 2^. To fabricate such films, various techniques, such as solution assembly, electrospining or template synthesis have been employed^3–7^. In contrast, physical vapor deposition (PVD) has showed some advantages of accommodating various types of sources, producing high-purity deposits, and fabricating large-area ordered films^8, 9^. Moreover, PVD is able to yield a series of kinetic products, which have diverse morphologies, by altering carrier gas flow rate, precursor temperature, and sample collection temperature^10–14^. The main challenge is that the films are generally difficult to lift off and transfer because of the strong material-substrate interaction. As a result, expensive substrates and low compatibility of special treatment limit their practical applications.

Free-standing films can be easily transferred onto the desired substrate to produce the final devices^3^. Such device assembling process can decrease remarkably the cost without sacrificing the device performance. However, special substrates and lift-off processes are needed in most cases. Thus, a feasible and low-cost method for the formation of free-standing organic films with ordered crystallinity is still challenging. On the other hand, ionic liquids (ILs) have attracted great attention due to their unique physical and chemical properties such as zero pressure of saturated vapor, extended temperature range, good chemical and thermal stability, and low flammability^15–18^. The application of ILs to vapor deposition is recently confirmed by our group^19^ and Matsumoto *et al*.^20, 21^. However, the products in previous studies are dispersed in ILs, and no continuous films are formed. It was because the hard control of the delicate balance of surface energy, interfacial energy and interaction between the target molecules.

In this study, free-standing films of metaloctaethylporphyrins (MOEPs) are directly grown for the first time by a PVD process on surface of an IL, which has higher density than MOEPs and is insoluble with MOEPs. The targeted MOEPs were well-known macrocyclic complexes and widely used in various fields such as chemistry, physics, biology, medicine, and device. They can be easily synthesized and have abundant properties, which can be elaborately adjusted via substitution by various groups^11, 22^. The monitoring of time-dependent process indicates that the strong π…π interaction between MOEP molecules and the high surface energy of IL played key roles in the formation of compact, plannar, and crystalline films. The good transferability of the as-obtained film makes it possible to be easily transferred to any substrates for further device application. The prototype photodetectors based on these films showed excellent flexibility, mechanical stability, and durability.

## Results

**Figure 1:**
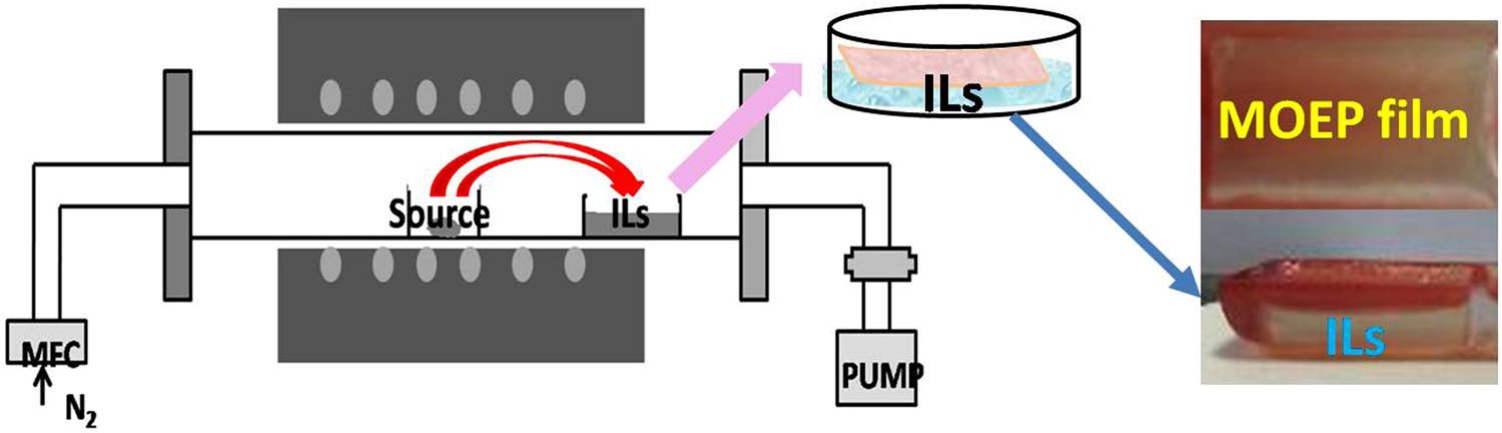
Schematic illustration of preparing and transferring process of MOEP free standing film.

Znic octaethylphorphyrin (ZnOEP) was hereinafter selected as a representative molecule to illustrate the morphology, structure, and growth mechanism of free-standing films. The ionic liquid of 1-(2-hydroxyethyl)-3-methylimidazolium tetrafluoroborate ([HEMIM][BF_4_]) was used as a medium for PVD process. Figure 1 shows a schematic illustration of the formation of free-standing films on surface of ILs. In brief, a small quantity of [HEMIM][BF_4_] was loaded by quartz boat and placed at the low-temperature in the horizontal tube furnace. After 30 min of deposition, free-standing film can be obtained on surface of [HEMIM][BF_4_]. It should be mentioned that, the density of IL is an important factor to form such free-standing film, which was unlike our early study of using 1-butyl-3-methylimidazolium tetrafluoroborate as substrate^19^. The area of films could be easily adjusted by varying the size of quartz boat and the quantity of starting materials. More importantly, the floating film can be easily transferred onto various solid substrates, such as Si, quartz, glass, and plastics (Fig. S1a~d).

**Figure 2:**
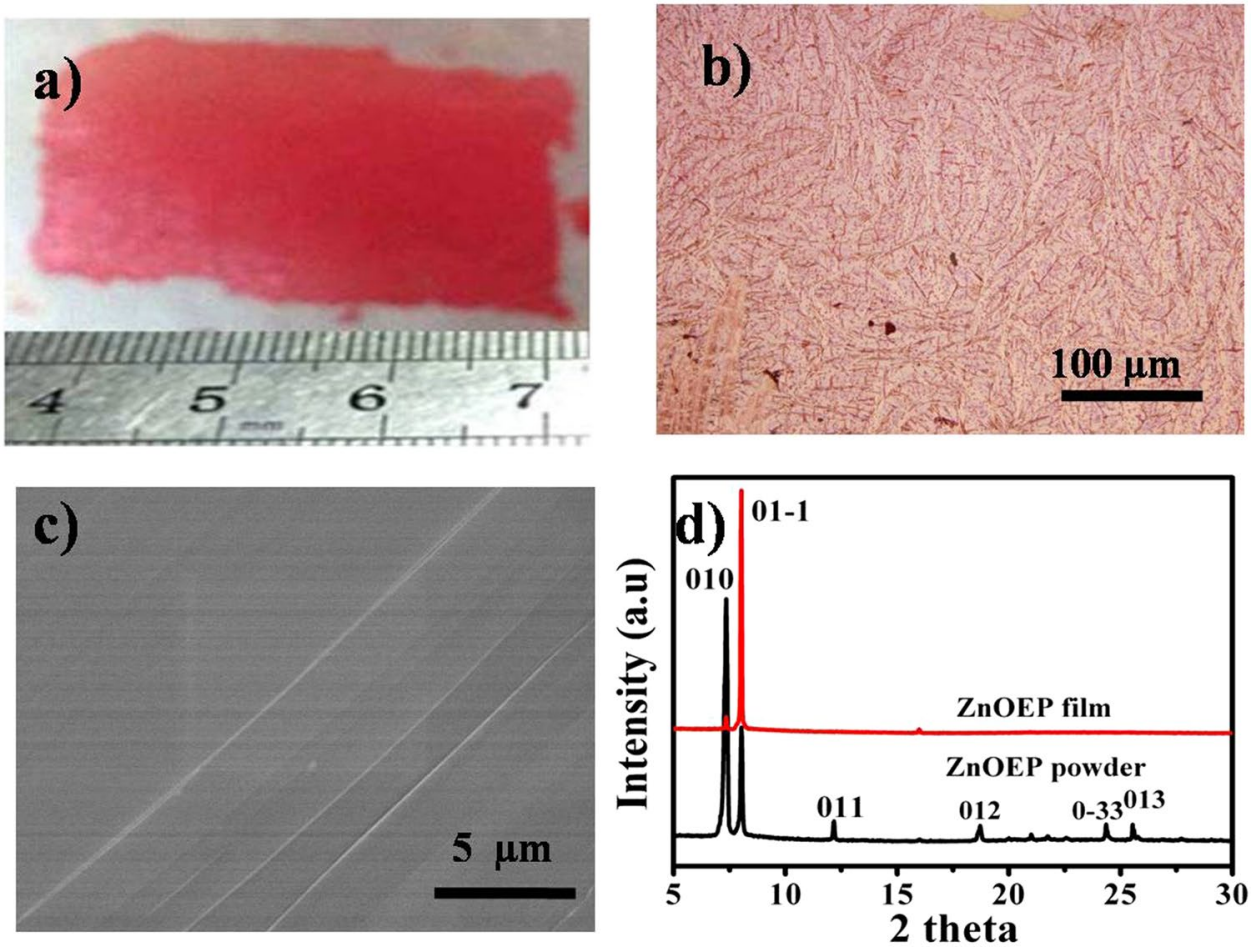
(**a**) Photographs of the typical free standing film and transferred onto the paper substrate. (**b**) The optical microscopic image of ZnOEP free standing film. (**c**) The SEM image of ZnOEP free standing film. (**d**) XRD patterns of the ZnOEP powder and ZnOEP film.

Figure 2a shows a photograph of a piece of free-standing film. The size of film was 3 cm × 1 cm, suggesting that it was possible to synthesize the free-standing film on a very large scale. Figure 2b shows an optical microscopic (OM) image of the as-obtained free-standing film. It can be seen that the film is very compact and made of many small cowry units, which are arranged into ordered structure and very smooth (Fig. 2c). The crystal structure of the film is examined by the X-ray diffraction (XRD) pattern as shown in Fig. 2d. The XRD patterns of the ZnOEP film are very sharp and consistent with that of the ZnOEP powder. The diffraction peaks are in good agreement with the literature and can be indexed as triclinic lattice^23^. The lattice constants are a = 4.692 Å, b = 13.185 Å, c = 13.28 Å, α = 113.94, β = 91.177 and γ = 92.175. Moreover, the diffraction peaks of (01–1) planes of the film are significantly enhanced relative to the ZnOEP powder. These results indicate that the film is highly crystalline and the preferential growth orientation is perpendicular to (01–1) plane.

**Figure 3:**
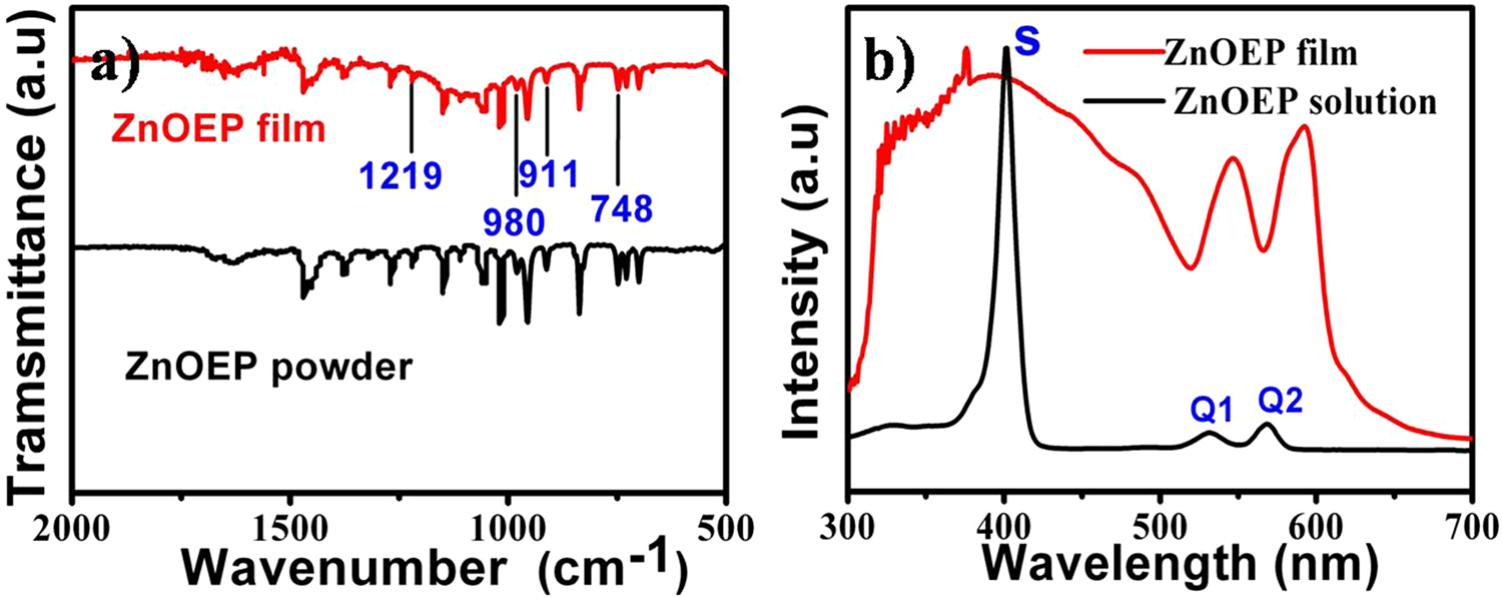
(**a**) FT-IR spectra of ZnOEP free-standing film (red line) and ZnOEP powder (black line). (**b**) UV-vis absorption spectra of ZnOEP free-standing film (red line) and ZnOEP monomers in cholroform solution (black line).

The Fourier transform infrared (FT-IR) spectra of both the film and the source powder of ZnOEP were recorded and shown in Fig. 3a to reveal the chemical composition of the film and possible structure change after vapor deposition. It can be found the FT-IR spectrum of the film has the same features as that of ZnOEP powder. Four metal-sensitive IR bands of octaethylporphyrin at 748, 911, 980 and 1219 cm^−1^ are very similar in both cases, which indicates that ZnOEP molecules did not undergo decomposition or other chemical reactions during the PVD process^24^. Figure 3b shows the ultraviolet-visible (UV-vis) absorption spectra of the film transferred onto quartz substrate and the ZnOEP monomers dissolved in chloroform. Similar to the monomers in solution, the film has three characteristic adsorption bands, labeled as S, Q1, and Q2. It is noted that all absorption bands are broadened in the film, in contrast to the sharp peaks in solution. Moreover, the Q bands of the film are red-shifted, whereas the S band of the film is blue-shifted. These changes of absorption spectrum are consistent with that reported by Bard *et al*., which can be attributed to highly ordered molecule-packing in the film^25^.

## Discussion

**Figure 4:**
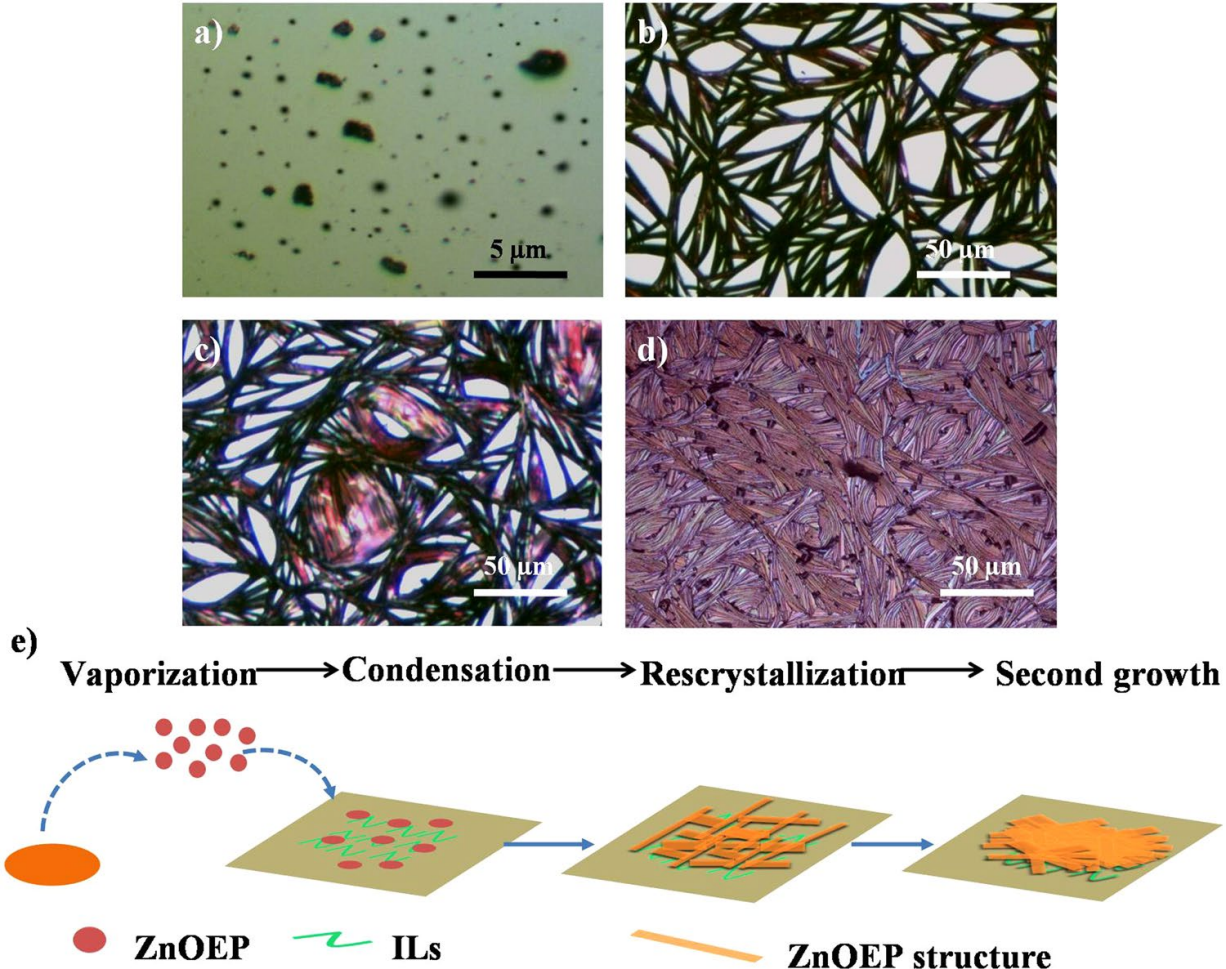
(**a**~**d**) The optical images of ZnOEP samples obtained at different deposition time after deposition temperature reached 325 °C: (**a**) 1 min, (**b**) 5 min, (**c**) 15 min, and (**d**) 30 min. (**e**) Schematic illustration of the formation of ZnOEP film.

The morphology evolution of ZnOEP film with different deposition times under the vaporization temperature of 325 °C was tested to reveal the growth mechanism. Figure 4a–d show a set of optical microscopic image of the film obtained at different deposition time. Many spherical particles (100–200 nm) together with few shaped pieces (up to 1 µm) in some regions are formed in 1 min (Fig. 4a). As deposition time goes on five minutes (Fig. 4b), the interconnected strips with tree trunk structure grow from certain positions on the ILs surface and form 2D networks instead of spherical particles. With the deposition time reaching 15 min, some thin flakes fill the vacancy between the cross-linked strips along the tree trunk (Fig. 4c). Finally, the above-mentioned strips and nanoflakes can connect closely with each other. After 30 min, the compact film with ordered structure was formed (Fig. 4d).

On the basis of the above observation, it can be assumed that the formation of the free-standing films under a vaporization-condensation-recrystallization-second growth mechanism and shown in Fig. 4e ^26–28^. The thermodynamic equilibrium structures of ZnOEP are determined by the balance between interfacial energy, the inter-molecule interaction and surface energy. At the early stage, the ZnOEP powder was vaporization into ZnOEP molecules and condensation into nucleation on the ILs surface. After that, with the strong π…π stacking between ZnOEP molecules, the multi-nucleation aggregates together to crystallization into one dimensional tree trunk structure floating on the ILs surface. Finally, the balance between surface energy (E_s_) and interfacial energy dominates the final products. In this case, the compact plannar structure could be easily obtained on the ILs surface compared with the solid substrate. This is because the second growth process mainly depends on the higher E_s_. The E_s_ of ILs is higher than solid and the super-saturation near the tree trunk is higher than the vacant place. As a result, supersaturated ZnOEP molecules could preferentially deposite on the ILs surface, which is between the interconnected tree trunk to fill the vacancy. It is because organic molecules could preferentially deposite on the higher Es sites^13^. This is obviously different from the vertical growth mode on the solid substrate.

**Figure 5:**
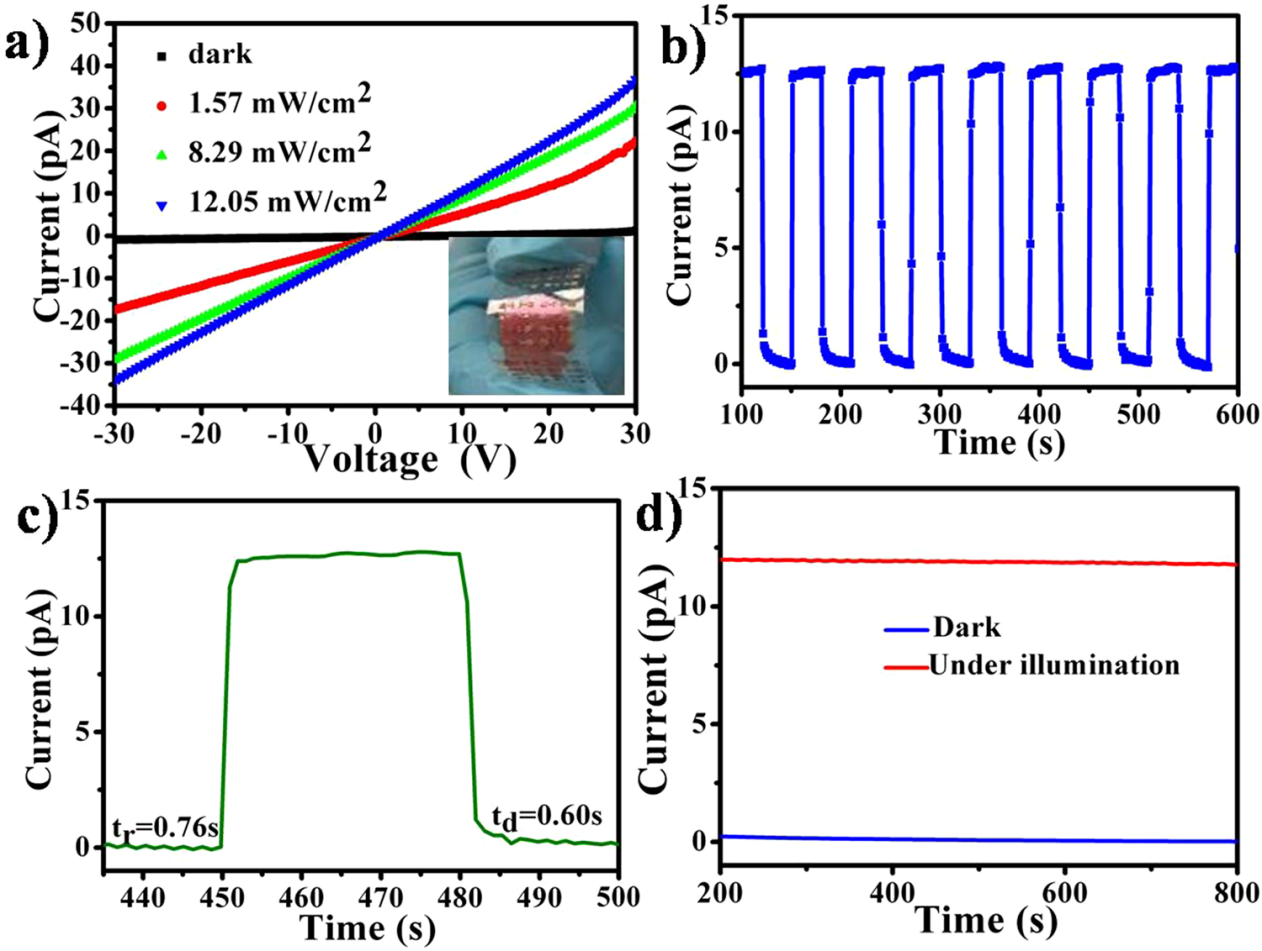
(**a**) *I-V* curves for ZnOEP based photodetector in the dark and under illumination with different intensity white light at an applied bias of 30 V. The inset is a typical image of the flexible photodetector device. (**b**) Photoresponses of the detector under a continuous 12.05 mWcm^−2^ white light rectangle pulse at an applied bias of 10 V. (**c**) Response time/recovery time of the device. (**d**) Current of the device measured in the dark and under white light illumination at a bias of 10 V.

The compatibility of such free standing film with substrates is important for the potential application in electrical device. Flexible prototype devices based on the free standing ZnOEP film were fabricated by combination of printing processes. ZnOEP film was transferred onto PET substrate with as-printed Ag eclectrode. The channel of electrode was 300 μm. The schematic prototype device based on ZnOEP film was shown in Fig. S2. The current voltage (I-V) curves of the device have been measured in the dark and under illumination with white light at different power intensities from 1.57 to 12.05 mWcm^−2^ and shown in Fig. 5a. From the curves, it can be seen that the dark current is close to zero with bias ranging from −30V to 30 V and the light current increases gradually with the increased light intensities at the same voltage. It indicates good photoresponse properties of the device. Furthermore, the *I–V* curves are linear and quasi-symmetric, indicating the excellent Ohmic contacts of the Ag electrodes with the ZnOEP film. A high current of 12.5 pA was recorded at an applied voltage of 10 V when the device was illuminated white light at a power intensity of 12.05 mWcm^−2^, while the dark current was only 0.03 pA, giving an Ion/Ioff ratio of 417. The photocurrent and on/off ration increased with increasing the thickness of the film (Fig. S3). The low current of the device may attribute to the large distance of the electrode. The anisotropy of photo-conductivity of ZnOEP films was studied with a sandwich device geometry (PET/ITO/ZnOEP/ITO/PET). The *I-V* curves for such photodetector in the dark and under illumination with different intensity white light at an applied bias from 30 V to −30 V was shown in Fig. S4. A high current of 1.1 nA was recorded at an applied voltage of 10 V when the device was illuminated white light at a power intensity of 12.05 mWcm^−2^, while the dark current was only 0.25 nA. It can be seen that the currents of sandwich device are much higher than the flat structure due to the large area connection of between ZnOEP and two electrodes. But the average on/off ratio is not exceed 10, which is much lower than the flat structure. As a result, we choose the flat structure for further study. Figure 5b displays the photocurrent transient measurement by periodically turning on and off white light with the power intensity of 12.05 mWcm^−2^ and at an applied voltage of 10 V. It can be found that the photocurrent increases rapidly and reaches steady state at the “ON” state under white light illumination and then decreases quickly to the “OFF” state after the light is turned off. The on/off ratio almost keeps the same after 8 cycles. The rise time and decay time, defined as the time taken for the initial current to increase to the 90% of the peak value, or vice versa, are measured to be 0.76 and 0.60 s, as shown in Fig. 5c. Furthermore, the device also exhibited a high stability under white light illumination. No obvious current decrease was observed after illumination of 600 s (Fig. 5d). The high sensitivity and fast photoresponse are attributed to chemical adsorption/desorption of oxygen on the ZnOEP film surface and barrier dominated conductance. In present study, the compactness and ordered cowry units structure of the ZnOEP film facilitated oxygen molecules adsorbed on the surface. This process creates a depletion layer with low conductivity and results in low dark current^29, 30^. Under the illumination of white light, the photogenerated holes moved to the surface and then along desorbed oxygen by band-bending. Hence, there is a significant current increase caused by the enhancing the separation of photo-generation carriers^31^. Due to the cowry unit construction of the compact film, we attributed the outstanding performance of the photoconductive devices to the fast carrier transport and the enhanced oxygen molecule adsorption of the film. The above observations all proved the promising potential of the free standing film device as a photodetector. More importantly, such growth-transfer separation is the key step to break through the limitation of PVD method on flexible thin film device.

**Figure 6:**
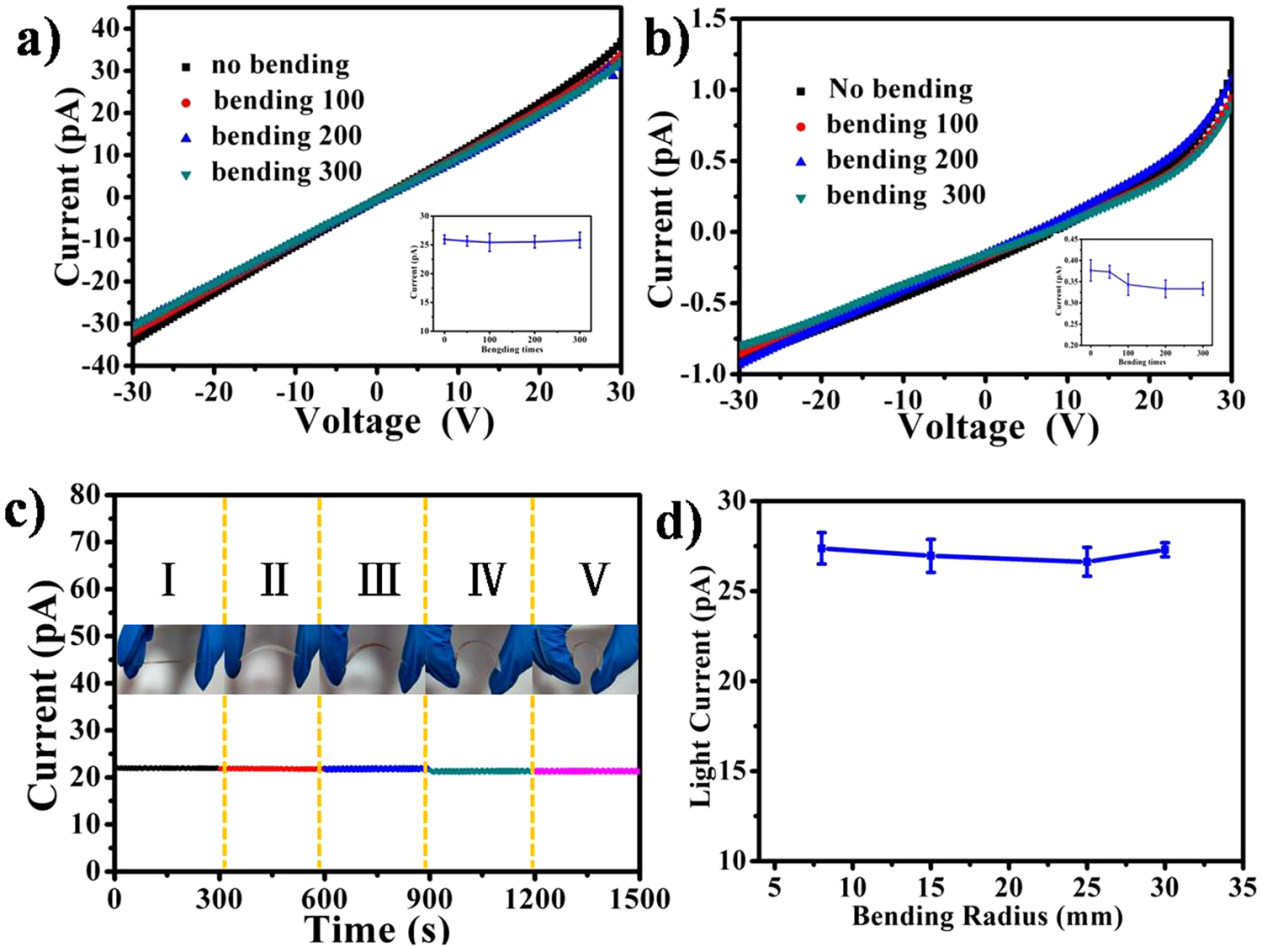
(**a**) *I-V* curves of flexible device without and after different bending cycles under a power irradiation of 12.05 mWcm^−2^ white light illumination. (**b**) The corresponding *I-V* curves in dark; Inset is corresponding the current error bar spectra of at a bias of 20 V. (**c**) Current of the flexible photodetector bent with various curvatures under the power of 12.05 mWcm^−2^ white light illumination at a bias of 20 V. The insets are corresponding photographs of the device under the different bending states. (**d**) Current of the flexible photodetector bent with different radii bending radius at a bias of 20 V.

In order to be fit for real applications, the electrical properties of the flexible devices under bending should remain unchanged. Figure 6a and b shows the *I-V* characteristics of the flexible device before and after bending for different cycles in dark and under the power of 12.05 mWcm^−2^ white light illumination. Compared with the flexible device without bending, the* I-V* curves showed nearly identical behavior even after 100, 200 and 300 cycles of bending. The device current increases gradually as the light intensity increases and still with good ohmic contacting behaviours even after 300 bending cycles in Fig. S5. In addition, the photocurrent at different curvature radiuses was marked at five different bending conditions, labeled as I, II, III, IV and V in the upper insets in Fig. 6c. It can be observed that the photocurrent kept unchanged at a fixed voltage of 20 V, revealling the fact that the conductance of the flexible device is hardly affected by the external bending stress. The excellent flexibility and mechanical stability were attributed to the compactness of film and good Ohmic contacts of the structure^32^.

**Figure 7:**
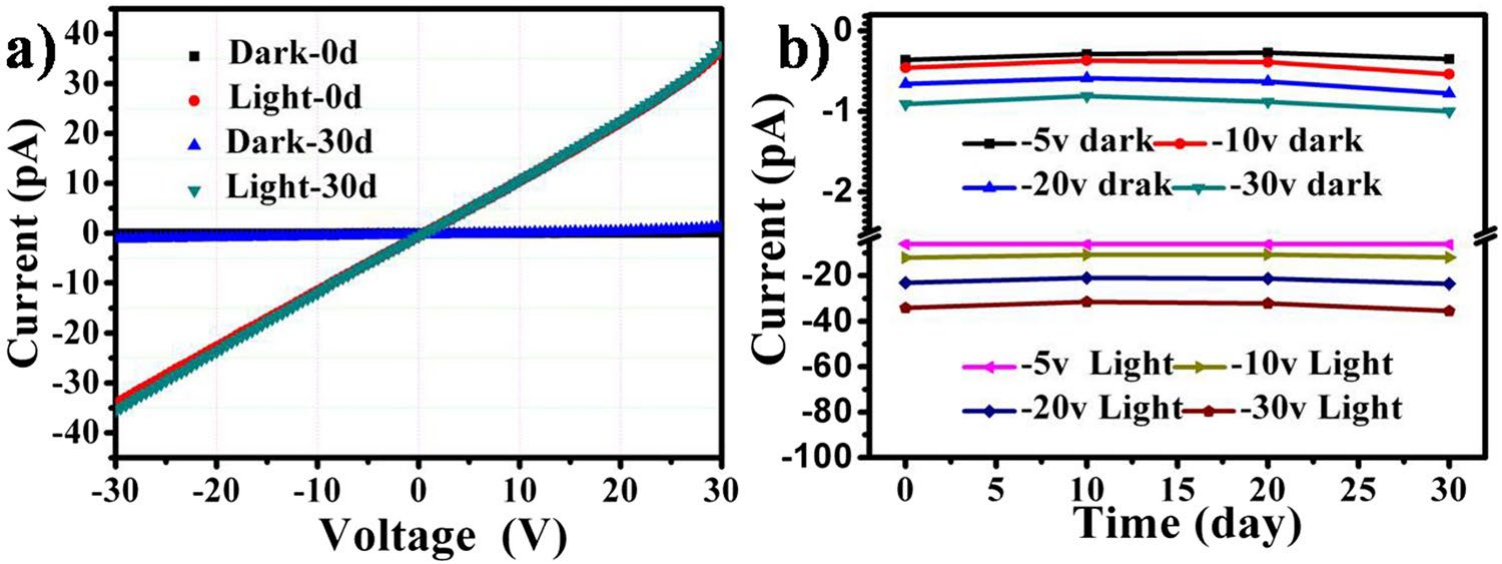
(**a**) *I-V* curves of the device measured in dark and under a power of 12.05 mWcm^−2^ white light illumination at 0 day and after 30 days. (**b**) The curves of the currents at different time.

The durability of flexible device is also another important factor in the real application. To determine the durability of the flexible device, the device was exposed to air without any encapsulation. The *I-V* curves of device in dark and under the power of 12.05 mWcm^−2^ white light illumination at 0 day and after 30 days was shown in Fig. 7a. The device still showed good photoelectrical property and is also with good ohmic contacting behaviour. The current almost remained unchanged even after 30 days in Fig. 7b, indicating the excellent device durability. These results indicate the high flexibility, good bending strength and electrical stability of the device. Compared to the photodetectors made by ZnOEP nanostructures obtained from other processes, the free-standing film reported here can be easily and conveniently transferred onto many substrates to producing flexible device and other desired devices in an environmental way without using any organic solvent^23, 33, 34^. The good transferable and good compatibility characteristics of such free standing film makes flexible devices ultra-mechanical stability and durability. The above observations proved that the free-standing film has a promising potential application in a highly photosensitive detector.

**Figure 8:**
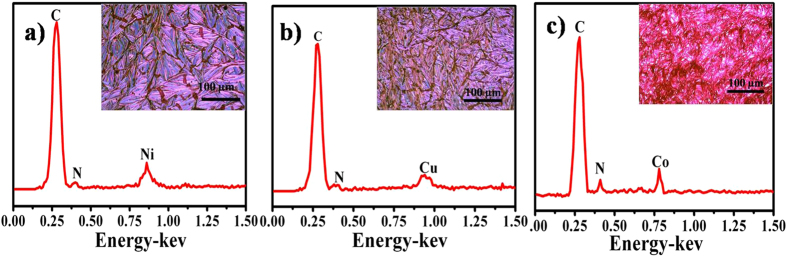
OM images of MOEPs (M = Ni, Cu, Co) free standing film and their corresponding EDX spectra.

Furthermore, it is important to point out that the present fabrication method can be applied to other MOEPs. Similar free standing films were also obtained for other MOEPs, such as CuOEP, CoOEP, and NiOEP. All the films of CuOEP, CoOEP, and NiOEP were made of many small cowry units, which are arranged into ordered flat structure. The delicate difference was that the densities of cowry units are difference from each other. This could be attributed to the different sublimation temperatures of MOEPs, which are induced by different intermolecular interaction between MOEP molecules, due to the incorporation of different metal ions. The recorded EDX spectrum (Fig. 8) of each film unambiguously shows the peak of the metal element (Ni, Cu, and Co, respectively), which originates from the metal center of the corresponding porphyrin molecules.

In summary, free-standing films of metal (Zn, Ni, Cu, Co) octaethylporphyrins were prepared for the first time by a PVD method on surface of [HEMIM][BF_4_]. Different from those on solid surfaces, the as-obtained MOEP films were very compact and with plannar structure. The monitoring of time-dependent process indicates that the high surface energy of [HEMIM][BF_4_] and the strong π-π interaction between MOEP molecules played key roles in forming such films. The outstanding transferability of such free-standing film has been confirmed by perfectly transferred such film onto various substrates such as Si, quartz, and PET. The prototype photodetectors based on free-standing films of ZnOEP shows ultra-mechanical stability and durability.

## Methods

### Materials

Metal (metal = Zn, Ni, Cu, Co) octaethylporphyrin(MOEP) were purchased from Sigma-Aldrich Chemical Co., Ltd. 1-(2-hydroxyethyl)-3-methylimidazolium tetrafluoroborate ([HEMIM][BF_4_]) was purchased from Shanghai Chengjie Chemical Co., Ltd. MOEPs and [HEMIM][BF_4_] were used without purification.

Preparation of MOEP free-standing film: In a typical procedure, 5 mg MOEP powder was loaded in a quartz boat, which was placed into a quartz tube inside a horizontal tube furnace. The MOEP was heated to 325 °C at a heating rate of 10 °C min^−1^. The protection of nitrogen was adopted during the process of vapor deposition to prevent the MOEP from being oxidized. The flow rate of nitrogen was kept at 100 standard cubic centimeters per minute. 3 ml [HEMIM][BF_4_] was loaded in a quartz boat and placed downstream by about 11 cm to collect the products.

### Characterization

The as-fabricated film was examined by scanning electron microscopy (SEM, Quanta 400 FEG), X-ray diffraction (XRD, X’Pert-Pro MPD), Fourier-transform infrared spectroscopy (FT-IR, Nicolet 6700), and ultraviolet-visible spectroscopy (UV-vis, Perkin-ElmerLambda750).

### Device fabrication and measurement

The photodetector device structure was constructed in a bottom-connected configuration. The patterned electrodes with a length of 1000 μm, width of 300 μm, and distance of 300 μm were fabricated by screen printing Ag on the PET substrate. ZnOEP film was rinsed with methanol to remove the residual ILs and then transferred onto the Ag electrodes. Current-voltage characteristics of the devices were recorded with a Keithley 4200 SCS and probe station in a clean and shielded box at room temperature. A xenon lamp was used as a white light source with different intensities. The intensity of the light was measured using a Coherent PowerMax. All measurements were carried out at room temperature under ambient conditions.

## Electronic supplementary material

Supplementary information
