# Supplementary material for: Direct physical vapor deposition and flexible photoelectrical properties of large-area free-standing films of metal octaethylporphyrin on ionic liquid surface
Source: Sci Rep. 2017 Aug 29;7:9838. doi: 10.1038/s41598-017-10293-2 (PMC5575091; doi:10.1038/s41598-017-10293-2)
Supplement: Supplementary file 1 — Supplementary information [file 41598_2017_10293_MOESM1_ESM.pdf]

Supporting Information

Direct physical vapor deposition and flexible photoelectrical properties of large-area free-standing films of metal octaethylporphyrin on ionic liquid surface

Yan Xiao,[1,2] Feng-Xia Wang, [1] Jia-Mei Yang, [1] Miao-Rong Zhang [1,2] and Ge-Bo Pan[1]*

†

[1] Suzhou Institute of Nano-tech and Nano-bionics, Chinese Academy of Sciences Suzhou 215125, Jiangsu, China.

[2] University of Chinese Academy of Sciences 100049 Beijing, P. R. China

*corresponding gbpan2008@sinano.ac.cn

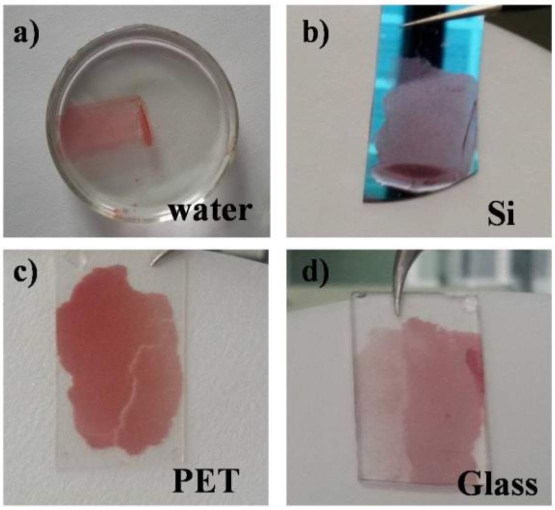

Figure s1. Photographs of ZnOEP free-standing film transferred onto different substrate. (a) water, (b)Si, (c) PET and (d) glass.

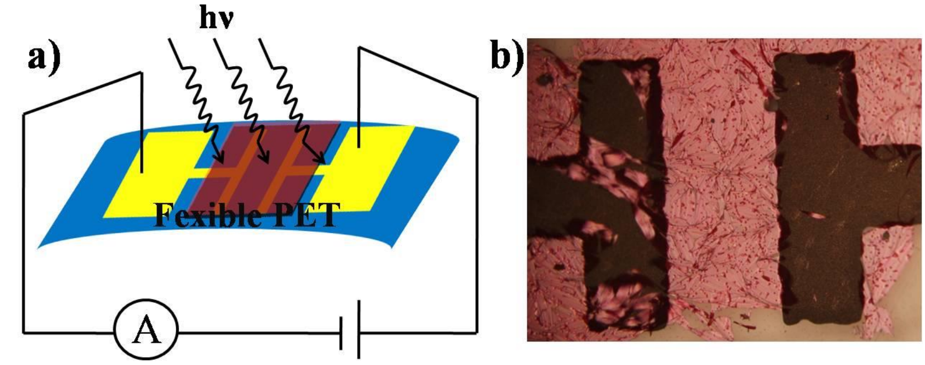

Figure S2. (a) Schematic illustration and (b) photograph of a typical device based on the ZnOEP free-standing film.

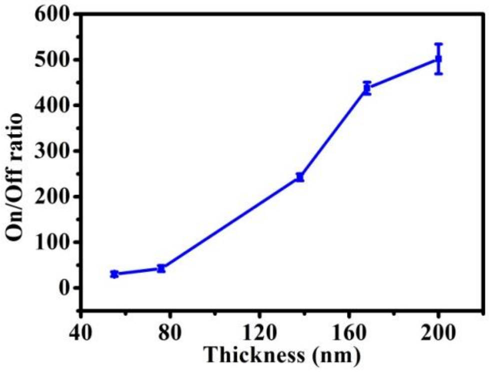

Figure S3. The on-off ratio with different thickness of ZnOEP film.

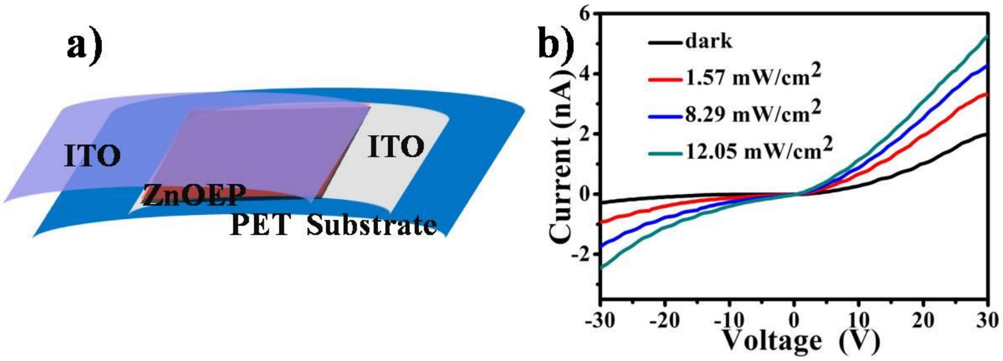

Figure S4. (a) Schematic illustration of thesandwich device (b) I-V curves for ZnOEP based sandwich photodetector in the dark and under illumination with different intensity white light at an applied bias of 30V.

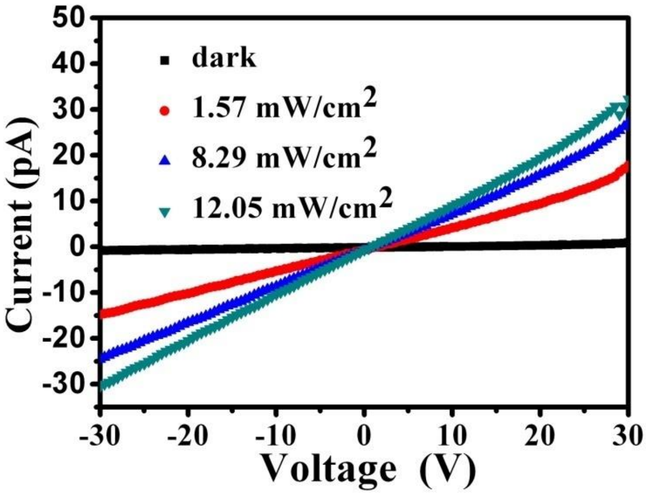

Figure S5 I–V curves of the flexible device measured in the dark and under illumination with different light intensities after bending 300 cycles.
